# Systematic review of outcome measures in pediatric eosinophilic esophagitis treatment trials

**DOI:** 10.1186/s13223-016-0144-y

**Published:** 2016-08-31

**Authors:** Tamar Rubin, Jacqueline Clayton, Denise Adams, Rabin Persad, Sunita Vohra

**Affiliations:** 1 Department of Pediatrics, University of Alberta, 11405-87 Avenue, 3rd Floor, Edmonton Clinic Health Academy, Edmonton, AB T6G 1C9 Canada; 2 CARE Program, University of Alberta, Suite #1702, College Plaza, 8215 112 St NW, Edmonton, AB T6G 2C8 Canada; 3 Department of Public Health Sciences, University of Alberta, 3-300 Edmonton Clinic Health Academy, 11405-87 Ave, Edmonton, AB T6G 1C9 Canada; 4 Department of Pediatric Gastroenterology and Nutrition, University of Alberta, 11405-87 Avenue, 3rd Floor, Edmonton Clinic Health Academy, Edmonton, AB T6G 1C9 Canada

**Keywords:** Eosinophilic esophagitis, Outcome measures, Pediatric, Systematic review, Treatment

## Abstract

**Background:**

Heterogeneity has been noted in the selection and reporting of disease-specific, pediatric outcomes in randomized controlled trials (RCTs). The consequence is invalid results or difficulty comparing results across trials. The primary objective of this systematic review was to assess primary outcome and outcome measure selection and reporting, in pediatric eosinophilic esophagitis (EoE) treatment trials. As secondary objectives, we compared trial disease definition to established concensus guidelines, and the efficacy of current EoE treatments.

**Methods:**

We searched MEDLINE, EMBASE, The Cochrane Library, Cochrane Central Register of Controlled Trials (CENTRAL), and CINAHL since 2001. We also searched clinical trial registries (portal.nihr.ac.uk; clinicaltrials.gov; isrctn.com; and anzctr.org.au) and references of included studies. We included RCTs of EoE treatment in patients 0–18 years. Two authors independently assessed articles.

**Results:**

Eleven studies met inclusion criteria. All identified primary outcomes, however, of 9 unique primary outcomes, only 2 were used in more than one study. In total, 25 unique primary and secondary outcome measures were employed for pediatric EoE treatment trials. Measurement properties and rationale for their selection was rarely provided. Uptake of consensus-based diagnostic criteria was 25 % in trials initiated after 2011. Due to the small number and heterogeneity of studies obtained, no meta-analysis of treatment efficacy could be undertaken. This SR was limited to exclusively pediatric RCTs.

**Conclusions:**

The results of this study confirm the need for a standardized set of core outcomes that are universally reported in pediatric EoE trials. Consistent disease definition and standardized outcome reporting will facilitate meta-analyses across similar trials and inform future clinical decision-making.

*Systematic review registration number* CRD42013003798

## Background

In randomized controlled clinical trials (RCTs,) the primary outcome is “the outcome of greatest importance,” [1] and is also the variable that determines calculation of the sample size. Outcome measures, in contrast, are the tools used to measure the primary outcome, and may be scales, questionnaires, scoring systems or other instruments [2, 3]. Although RCTs are universally recognized as the gold standard for determining treatment efficacy, the validity of their results depends on the selection of the most appropriate primary outcomes, valid outcome measurement instruments, and full reporting of the originally stated primary outcomes [4].

A more standardized approach to the selection of outcome measures for disease-specific pediatric RCTs has been proposed as one strategy to help facilitate knowledge synthesis [5]. Standardized outcome selection and reporting, regardless of statistical significance, might also minimize outcome reporting bias [6]. Selective outcome reporting is now well accepted as a significant impediment to knowledge translation and meta-analysis [7]. To this end, initiatives such as the Consolidated Standards of Reporting Trials (CONSORT) have been established to help promote transparent and complete reporting [1, 8].

In order to facilitate outcome measure selection, the consensus-based standards for the selection of health measurement instruments (COSMIN) group developed an international consensus on the terminology and definitions of measurement properties [9]. They identified three domains of measurement properties: reliability, validity, and responsiveness. Other international initiatives aiming to improve selection and reporting of outcome measures include the COMET initiative (Core Outcome Measures in Effectiveness Trials), which is an initiative to develop a core set of outcome measures for each condition [4].

Methods for appropriate selection of outcome measures in clinical trials have been studied, to some extent, in adults, but very few studies have addressed this problem in children [3]. The validity of outcome measures chosen in pediatric RCTs, as well as the adequacy of their reporting, has been called into question [2, 3, 5, 10]. A recent systematic review (SR) of pediatric RCTs found that more than 10 years after CONSORT guidelines were developed, 25 % of pediatric RCTs published in high impact journals still failed to identify a primary outcome [11]. Furthermore, measurement properties of outcome measures were often not reported. Other systematic reviews within specific clinical subspecialities have identified similar problems [12–14].

In order to examine the issues surrounding outcome measure selection and reporting in pediatric RCTs in greater depth, a systematic review within a clinical subspecialty of pediatrics was planned. Eosinophilic esophagitis (EoE) is an immune-mediated inflammatory disease of the esophagus defined by symptoms of esophageal dysfunction and histopathologic findings. This particular condition was strategically chosen as it is a relatively new condition, where many RCTs on the topic would be expected to have been designed well after the development of COSMIN and CONSORT guidelines. Furthermore, heterogenous disease definition in EoE was identified relatively early on, as being an issue in the EoE literature [15]. In 2007, in order to address some of these concerns, the First International Gastrointestinal Eosinophil Research Symposium (FIGERS) published consensus guidelines to help improve treatment and diagnosis of EoE (Table 1) [16]. By 2011, newer updated guidelines, including a revised “conceptual definition” of EoE was developed [17].

**Table 1:** FIGERS criteria for EoE [17]

1	Clinical symptoms of esophageal dysfunction (in infants and small children, GERD-like symptoms and feeding problems; in older children and adults, GERD-like symptoms, especially dysphagia or esophageal food impaction)
2	≥15 eosinophils in at least one high-power field and;
3	Either lack of histological response to 6–8 weeks of treatment with high-dose proton-pump inhibitor, OR a normal pH monitoring study of the distal esophagus

The most recent definition states that EoE is a “chronic, immune/antigen-mediated esophageal disease characterized clinically by symptoms related to esophageal dysfunction and histologically by eosinophil-predominant inflammation” [17]. This definition, and its accompanying diagnostic guidelines, emphasizes that both clinical features of esophageal dysfunction, and pathologic features of the disease must be present. The presence of ≥15 Eo/HPF in at least one endoscopic esophageal mucosal biopsy and/or the presence of other microscopic features of eosinophilic inflammation is required for diagnosis. As well, in order to exclude children with PPI-responsive esophageal eosinophilia, an 8-week trial of PPI prior to diagnosis is now also recommended.

The 2011 revisions were a response to certain somewhat arbitrary requirements in the original definition (e.g. histologic finding of 15 or more Eo/HPF), which carry no proven biologic significance or power to discriminate amongst the various esophageal diseases. The requirement to rule out GERD (either via failure of PPI treatment or a normal PH impedance study) had not been rigorously applied to subsequent studies, nor validated. Furthermore, no studies were published since the original consensus report that could allow diagnosis based on a pathognomonic clinical/histologic feature or biomarker.

### Objectives

This SR assessed the heterogeneity of outcome measure selection and reporting in exclusively pediatric EoE treatment RCTs. As secondary objectives, this SR assessed the heterogeneity of definitions of EoE pre- and post-FIGER publication and evidence for current acute treatment modalities for EoE in the pediatric population.

## Methods

This review was registered on PROSPERO prior to the start of the study (CRD42013003798). The search strategy was developed in conjunction with a clinical health research librarian.

### Data sources

We searched MEDLINE, EMBASE, Cochrane Database of Systematic Reviews, Cochrane Central Register of Controlled Trials (CENTRAL), and CINAHL, using all terms relating to EoE. The search was limited to English-language studies published between January 2001 and December 5, 2014. We also screened the reference lists of included studies and searched selected websites for ongoing/registered trials including https://www.portal.nihr.ac.uk/Pages/NRRArchive.aspx; http://www.clinicaltrials.gov/; http://www.isrctn.com/; and http://www.anzctr.org.au/.

### Study selection

Studies were selected if they were: (i) RCTs or controlled trials; (ii) were restricted to pediatric patients (0–18 years) with EoE; (iii) investigated any modality used to treat EoE (e.g. steroids via any route of administration; immune modulating treatment, mast cell inhibitors, monoclonal antibodies; dietary manipulation; esophageal dilatation; novel modalities); and (iv) compared treatment to any control (including, but not limited to, placebo).

Two reviewers (TR, JC) independently screened the abstracts and/or full text of identified articles to determine which ones met criteria. Disagreement was resolved through discussion, including with a senior reviewer (DA, SV) as needed.

### Data collection and analysis

Full texts of all included studies were obtained. Data from included studies were independently extracted by the two reviewers. Disagreement was resolved through discussion, including with a senior reviewer as needed. The following information was extracted: journal name, publication year, design of RCT/CCT, sample size, intervention of interest, number of primary outcomes, outcome measures used, and details of outcome measurement properties. Flexibility in terminology to express “primary outcome” was allowed (e.g. main outcome, primary outcome, end-point etc.). Primary outcomes were examined in detail in order to identify their measurement properties. Information about safety and harms reporting was also extracted.

### Assessment of methodological quality of included studies

The two reviewers independently assessed included studies for risk of bias based on the cochrane risk of bias tool (http://www.cochrane-handbook.org). Where possible, study authors were contacted for additional information. While risk of bias assessment was not necessary to meet the primary objective of our review, it was useful when interpreting data regarding treatment effect.

## Results

**Fig. 1:**
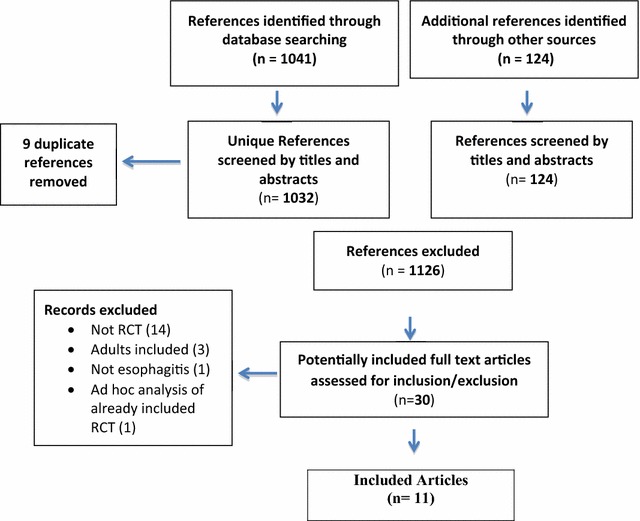
Flow diagram

A total of 1032 unique references were identified through database searches and another 124 from trial registries. Screening of titles and abstracts excluded 1126 references. Thirty full text articles were obtained and eleven met all inclusion criteria (see Fig. 1). Six were published studies [18–23] and five were registered trial protocols [24–28].

Table 2 provides an overview of the primary outcome measures selected and reported in these studies.

**Table 2:** Primary outcome measures selected and reported in pediatric EoE trials

	Konikoff [18]	Schaeffer [19]	Dohil [20]	Assa’ad [21]	Spergel [22]	Gupta [23]	Page [25] NCT01458418	Di Nardo [26] NCT01846962	Teva Pharm [27] NCT00635089	McGuire Davis [28] NCT01821898	Heine [24] ACTRN12613001210763
Esophageal eosinophilia or histologic remission (eosinophils/HPF)	X		X	X	X		X			X	X
Biopsy Grade		X									
Compound histologic and symptom response						X					
Physician Global assessment score					X						
EoE clinical symptom score						X					
Clinical severity score								X			
Safety				X					X		
Tolerability				X							
Pharmacokinetics				X							

Table 3 provides an overview of the secondary outcome measures selected and reported in these studies.

**Table 3:** Secondary outcome measures selected and reported in pediatric EoE trials

	Konikoff [18]	Schaeffer [19]	Dohil [20]	Assa’ad [21]	Spergel [22]	Gupta [23]	Page [25] NCT01458418	Di Nardo [26] NCT01846962	Teva Pharm [27] NCT00635089	McGuire Davis [28] NCT01821898	Heine [24]
_Esophageal eosinophilia_				_X_							_X_
_Endoscopic features_	_X_			_X_							
_Modified endocscopy tool_			_X_								_X_
_Histologic features_	_X_			_X_							
_Histology Scoring Tool_			_X_								
_Severity score for endoscopy and histology_								_X_			
_Clinical symptoms_	_X_										
_Predominant symptom assessment scores (patient report)_					_X_						
_“Clinical Response” (combined patient report and physician assessment)_		_X_									
_Symptom scoring tool_			_X_								_X_
_EoE symptom scoring tool_											
_Pediatric EoE symptom severity module_										_X_	
_CHQ scores_					_X_						
_PedsQL_										_X_	
_Genotypic features_			_X_								
_Blood eosinophil counts_				_X_							
_Immune features_										_X_	_X_
_Proteonomic features_							_X_			_X_	_X_
_Profile of durability of response to treatment_									_X_		
_Adverse events_					_X_						

### Outcome measures

All 11 included studies identified at least one primary outcome with 8 identifying a single primary outcome (Table 2). The number of primary outcomes per study ranged from 1 to 4.

Nine different primary outcome measures were found and only two were used in more than one study: esophageal eosinophilia (used in 8/11 studies) and safety (used in 2/11 studies). Although many of the studies attempted to assess similar types of primary outcomes (e.g. outcomes that included clinical symptoms), the outcome measures they selected varied: e.g. “physician global assessment score,” “EoE clinical symptoms score,” “clinical severity score.”

Of the 20 different secondary outcomes (Table 3), only 8 occurred in more than one study: Esophageal eosinophilia (n = 3), histologic features (n = 3), proteonomic features (n = 3), endoscopic features (n = 2), modified endoscopy tool (n = 2), symptom scoring tool (n = 2), immune features (n = 2), and adverse events (n = 2). Just as in the primary outcome measures, similar endpoints were often being assessed but used different measurement tools. For example, endoscopic features were assessed in at least 4 studies, however outcome measures were different and included “endoscopic features,” “modified endoscopy tool,” and “severity score for endoscopy and histology.” Likewise, symptoms were assessed in multiple studies (at least 6) but used a variety of outcome measures (e.g. “patient symptom report,” “clinical response,” “symptom scoring tool,” “predominant symptom assessment score,” and “pediatric EoE symptom severity module”).

Combining both primary and secondary outcomes (Table 4), there were 26 unique outcome measures and still only 8 that were used in more than one study: esophageal eosinophilia (n = 9), safety (n = 4), symptom scoring tool (n = 3), histologic features (n = 3), proteonomic features (n = 3), endoscopic features (n = 2), endoscopy scoring tool (n = 2) and immune features (n = 2). Nevertheless, several different outcome measures were often chosen to assess a similar outcome. For example, 9 distinct outcome measures were used to assess clinical symptoms, and at least 4 different outcome measures were used to assess histologic features.

**Table 4:** Combined primary and secondary outcome measures selected and reported in pediatric EoE trials

	Konikoff [18]	Schaeffer [19]	Dohil [20]	Assa’ad [21]	Spergel [22]	Gupta [23]	Page [25] NCT01458418	Di Nardo [26] NCT01846962	Teva Pharm [27] NCT00635089	McGuire Davis [28] NCT01821898	Heine [24] ACTRN12613001210763
Esophageal eosinophilia or histologic remission (eosinophils/HPF)	X		X	X	X	X	X			X	X
Histologic features	X			X		X					
Histology scoring tool			X								
Severity score for endoscopy and histology								X			
Biopsy grade		X									
Endoscopic features	X			X							
Modified endoscopy tool			X								X
Compound histologic and symptom response						X					
Clinical symptoms	X										
“Clinical response” (combined patient report and physician assessment)		X									
Clinical severity score								X			
Symptom scoring tool			X								X
EoE clinical symptom score						X					
Pediatric EoE symptom severity module										X	
Predominant symptom assessment scores (patient report)					X						
Physician global assessment score					X						
PedsQL										X	
CHQ scores					X						
Tolerability				X							
Pharmacokinetics				X							
Genotypic features			X								
Blood eosinophil counts				X							
Proteonomic features							X			X	X
Immune features										X	X
Profile of durability of response to treatment									X		
Safety or adverse events				X	X	X			X		

Of the total of 9 unique primary outcomes, 5 (56 %) were scales, scoring systems, instruments, questionnaires or other scoring tools. Of the 25 total primary and secondary outcome measures, 15/25 (60 %) were scales, scoring systems, instruments, questionnaires or other scoring tools. Table 5 summarizes the outcome measurement tools described.

**Table 5:** Outcome measurement tools and reporting of measurement properties

Outcome	Outcome measure	Measurement properties reported	Rationale for selection	Authors’ citations for measurement properties
Response of histologic features	Histology scoring tool	Non-validated	Previously reported tool	[40]
Response of histologic features and esophageal eosinophilia	Biopsy Grading System (numeric score based on composite of histologic features and esophageal eosinophilia)	Not reported	Review of available literature	[41–45]
Response of histologic and endoscopic features	Severity Score for Endoscopy and histology	(no data in trial listing)	(no data in trial listing)	–
Response of endoscopic features	Modified endoscopy tool	Non-validated	Previously reported tool	[40]
Compound histologic and symptom response	Combination of “EoE clinical symptom score” (CSS) and peak Eo ≤6/HPF in all esophageal levels. CSS is based on physician’s assessment of frequency and disruptiveness of multiple symptoms within 6 categories, and use and disruptiveness of coping behaviours, determined by questioning subject and/or caregiver	Non-validated	A newly developed symptom score adapted from a previously reported tool. Although symptoms evaluated are not EoE specific, the wide range of common symptoms reported in pediatric EoE patients of varying ages are included	[16, 17, 19, 37, 46]
Clinical response	Presence or absence of the presenting symptom by patient/guardian report and by physician assessment at predetermined intervals	Not reported	None provided	No reference provided
Clinical severity score	Each symptom scored on frequency, intensity, and interference on life quality. One point added in the presence of feeding difficulties leading to growth delay (weight/height ratio <5 %) or significant weight loss (>10 % of initial body weight). Two points added the in case of gastrointestinal bleeding or severe strictures requiring urgent hospitalization	No data in trial listing	No data in trial listing	
Symptom scoring tool	Symptom scoring tool, devised originally for children with acid peptic disorders	Not reported	The SST “is used regularly in our clinic” and has been used to differentiate between EoE, GERD and patients with other atopic and nontopic disorders	[40, 46–48]
EoE Clinical symptom Score	Newly developed, symptom score adapted from a previously reported scoring tool	Non-validated	“Although the symptoms evaluated are not EoE specific, the wide range of common symptoms reported in pediatric EoE patients of varying ages are included “	[19, 46]
Pediatric EoE symptom severity module	Measure of % of normal bolus transit swallow and % of normal peristaltic esophageal body contractions	No data in trial listing	No data in trial listing	
Predominant symptom assessment scores	Symptom with greatest negative effect on patient at baseline (based on 5-point rating) was followed throughout the trial.	Not reported	Not reported	No references provided
Physician EoE global assessment score	Taking into account clinical findings and patient report of symptoms, physicians rated severity of patient’s EoE as “none”, “mild,” “moderate,” “severe” or “very severe”	Not reported	Not reported	No references provided
CHQ Scores	CHQ	Not reported	Not reported	[49]
Quality of Life	PedsQL	No further description in trial listing	No further description in trial listing	–
Profile of durability of response to treatment	No further description in trial listing	No further description in trial listing)	No further description in trial listing)	–

The measurement properties for chosen instruments were only reported in 4/15 cases, and all were unvalidated. Reliability and responsiveness were never reported. Rationale was provided for selection of outcome measures in 6/15 cases, and included “prior use/reporting of these instruments,” “review of available literature,” and “ability of the tool to capture a wide range of symptoms specific to pediatric EoE.” References were provided for 7/15 of the outcome measurements.

### Esophageal eosinophilia

Although esophageal eosinophilia is a requisite component of the definition of EoE, not all studies measured this as an outcome. Furthermore, outcome measures of esophageal eosinophilia varied significantly in their definitions, methods of measurement, and time period of assessment. For example, some studies defined histologic remission as ≤1 Eo/HPF, while other defined remission as ≤5 Eo/HPF or 0–6 Eo/HPF. Other studies assessed percent change in peak Eo counts as evidence of treatment efficacy.

### Clinical symptoms

Clinical symptoms are also a requisite feature of the disease, and were often a component of outcome measurement tools. However, measurement of clinical symptoms in these studies also varied significantly. For example, some of the studies measured combined patient/parent and physician assessments, and others focused on one or the other. Various symptom-scoring tools were also employed.

### Disease definition

Of the 11 trials, 8 were initiated after the publication of the FIGERS criteria. Four out of these eight trials (50 %) met the original FIGERS criteria in their definition of EoE (Table 6). The study by Spergel et al. (and the extension study by Teva Pharmaceuticals) were considered to have met the FIGER criteria for defining EoE, although they accepted patients who had failed a 4 week trial of PPI (rather than the 6–8 week required trial outlined in the definition). Of the 4 trials that did not meet FIGER criteria, reasons were: no requirement for symptoms (2), and no requirement for a negative pH probe or PPI trial (2). Excluding the Spergel trial and its extension study would mean only 2/6 studies met criteria (33 % uptake).

**Table 6:** Summary of disease definition across trials

	Konikoff [18]	Schaefer [19]	Assa’ad [21]	Dohil [20]	Spergel [22]	Gupta [23]	Teva Pharm [27] NCT00635089	Page [25] NCT01458418	Di Nardo [26] NCT01846962	McGuire Davis [28] NCT01821898	Heine [24] ACTRN12613001210763
Initiated after 2007?	No	No	No	Yes (2008)	Yes (2008)	Yes (2008)	Yes (2008)	Yes (2011)	Yes (2013)	Yes (2013)	Yes (2013)
Met FIGER criteria?	N/A	N/A	N/A	Yes	Yes^a^	No	Yes^b^	No	Yes	No	No
Reason for not meeting criteria	No acid suppression or symptoms required		No symptoms required			No pH test or PPI trial required		No symptoms required		No symptoms required	No pH test or PPI trial required
Initiated after 2011?	No	No	No	No	No	No	No	Yes	Yes	Yes	Yes
Meet updated consensus criteria?	N/A	N/A	N/A	N/A	N/A	N/A	N/A	No symptoms indicated, no requirement for failed PPI trial	yes	No requirement for failed PPI trial for 8 weeks, requirement for >20 Eo/HPF	No requirement for failed PPI trial for 8 weeks, requirement for >20 Eo/HPF

^a^Only 4 week trial of PPI accepted

^b^Extension of Spergel study [22]

Four studies were initiated after publication of the updated EoE consensus guidelines. Only 1 out of 4 (25 %) met the new definition for EoE. Three of the studies did not require patients to have failed an 8-week trial of PPI (as suggested in the new guidelines), two required a more stringent definition of esophageal eosinophilia (>20/HPF) than required in the guidelines, and one did not require symptoms.

### Treatment efficacy

Six studies had results available (Table 7). Four studies measured the effect of swallowed steroid [18–20, 23], while 2 studies measured the effect of intravenous anti-IL5 therapy [21, 22].

**Table 7:** Histologic remission (Peak Eo count ≤1 in all fields in proximal and distal esophagus at 3 months)

Study	Design	Intervention	Control	1° Outcome(s)	2° Outcome(s)	Outcome reporting	Outcome reporting bias	Conclusions
Konikoff [18]	Randomized, double blind, 2 arm	Swallowed fluticasone propionate (440 μg BID × 3 months)	Placebo	Histologic Remission (Peak Eo count ≤1 in all fields in proximal and distal esophagus at 3 months)	a. Presence of endoscopic furrowing	1° Outcome: Yes	Some selective outcome reporting: Not all symptom data available for 2° outcome	Swallowed fluticasone effective in inducing histologic remission in EoE, with more pronounced effect in non-allergic and younger individuals, especially in proximal esophagus
					b. Reduction in epithelial hyperplasia	Post-hoc, histologic responsiveness also re-defined as higher peak Eo count; mean Eo count of <1 or <2; and % reduction of 90 or 95 %	Additional outcomes reported (Intervention responsiveness- allergic vs non-allergic subjects, age, height, weight; CD8+ T cell levels, esophageal mastocytosis, adverse events)	
					c. Presence of symptoms	2° Outcomes: a. Yes, b: Only vomiting reported		
						Harms: adverse events reported		
Schaefer [19]	Randomized, open label, 2 arm	Swallowed fluticasone (220 μg QID if 1–10 years, or 440 μg QID if 11–18 years) × 4 weeks with 8 week weaning protocol	Oral prednisone 1 mg/kg/dose BID (max 30 mg BID) × 4 weeks with 8 week weaning protocol	Improvement in “biopsy grade” by 1 or more after 4 weeks	Clinical response at 0, 4, 12, 18–24 weeks, based on presence or absence of presenting symptoms by patient report, and physician assessment.	1° Outcome: yes	Additional outcomes reported (proportion of symptom free patients, relapse rate, time to relapse, systemic adverse effects)	Systemic and topical corticosteroids effective in achieving initial histologic and clinical improvement. Prednisone resulted in greater degree of histologic improvement, without evidence of associated clinical advantage over fluticasone for symptom resolution, relapse rates or time to relapse. Symptom relapse common to both groups upon therapy discontinuation
						2° Outcome: yes		
						Harms: adverse events reported		
Dohil [20]	Randomized, double-blind, 2 arm	Oral viscous budesonide (<5ft: 1 mg; >5ft: 2 mg) + PPI	Placebo + PPI	Baseline and final peak esophageal Eo count/HPF: responders (0–6 Eo/HPF); partial responder (7–19 Eo/HPF); non-responders (≥20 Eo/HPF)	a. Endoscopic features (modified endoscopy tool)	1° Outcome: yes	None evident	OVB improves symptoms and endoscopic and histologic features of pediatric EoE compared to PPI alone
					b. Symptom response (symptom scoring tool)	2° Outcomes: yes		
					c. Histologic features (histology scoring tool)	Harms: adverse events reported		
					d.TGFβ1/TGFβ1 promoter genotype			
Assa’ad [21]	Randomized, double blind, 3 arm	Mepolizumab 2.5 or 10 mg/kg at day 0, week 4 and 8	Mepolizumab 0.55 mg/kg at day 0, week 4 and 8	a. Proportion of “responders” (peak Eo <5/HPF at week 12), % partial responders: 5–19 Eo/HPF	a. Changes in peak & mean Eo counts	1° Outcome: Yes	Post-hoc analysis of predictors of response for change in mean Eo counts.	IL5 involved in pathogenesis in EoE in children. mepolizumab reduces esophageal Eo inflammation in these patients
				b. Safety	b. Histopathologic findings	2° Outcomes: Yes	Additional outcomes reported: changes in esophageal Eo density, changes in symptoms	
				3. Tolerability	c. Endoscopic findings	Harms: adverse events reported		
				4. Pharmaco-kinetics	d. Blood Eo counts			
					e. Frequency + severity of symptoms			
Spergel [22]	Randomized, double blind, 4 arm	Reslizumab 1, 2 or 3 mg/kg infusions at 0, 4, 8 and 12 weeks	Placebo	a. Histologic: % change in peak esophageal Eo count	a. CHQ scores	1° Outcome: yes	Certain data selectively described (e.g. positive trends in patient predominant symptom assessment (secondary outcome), but not actual values	Reslizumab significantly reduced intraepithelial esophageal Eo counts in children and adolescents with EoE. However, improvements in symptoms were observed in all treatment groups and were not associated with changes in esophageal Eo counts
				b. Symptoms: Physician global assessment score week 15	b. Predominant symptom assessment scores	2° Outcomes: yes		
					c. Adverse events	Harms: adverse events reported		
Gupta [23]	Placebo-controlled, 4 am	Oral budesonide suspension: low-dose (0.35 mg if 2–9 years; 0.5 mg if 10–18 years), medium-dose (1.4 mg if 2–9 years; 2 mg if 10–18 years), high dose (2.8 mg if 2–9 years; 4 mg if 10–18 years)	Placebo	Compound histologic and symptom response to therapy	a. Percentage of subjects with histologic response (peak Eo ≤6/HPF)	Yes	None observed	Peak Eo counts significantly reduced throughout esophagus in pediatric patients with EoE given medium dose and high dose OBS. There was a large symptom response to placebo that was similar to symptom responses in the OBS groups. Symptom response did not distinguish OBS from placebo
					b. Clinical symptom response (≥50 % reduction in EoE clinical symptom score)	Harms/adverse events reported.		
					c. Histologic remission (peak Eo ≤1/HPF at all levels)			
					d. Symptom resolution (EoE CSS 0)			
Page [25] NCT01458418 (not yet recruiting)	Randomized, double-blind, 3 arm	Montelukast 10 or 5 mg/day	Placebo	Eo/HPF in the esophagus after 12 weeks of therapy.	Amount of MBP, tryptase and trichrome in esophageal specimens at 1–2 weeks	N/A	N/A	
Di Nardo [26] NCT01846962 (recruiting)	Randomized, open-label, 3 arm	Swallowed budesonide 0.5 mg	N/A	Clinical severity score (frequency, intensity and interference on life quality)	Severity Score for endoscopy and histology	N/A	N/A	
		Swallowed fluticasone 0.5 mg						
		6-food ED + triggering foods per patients or parents						
Teva Pharm [27] NCT00635089 (completed)	Open-label extension study for patients who completed Spergel study [22]	Reslizumab monthly	N/A	Safety	Profile of durability of response to treatment	Data not available	Data not available	
McGuire Davis [28] NCT01821898 (recruiting)	Randomized, 4 arm	Group 1 (+FA): sham ED + PO budesonide (1–2 mg based on weight)	N/A	Eo/HPF after treatment	a. Quality of Life at 8 weeks (PedsQL)	N/A	N/A	
		Group 2 (+FA): ED + sham PO budenoside			b. Symptom score (validated tool “pediatric EoE symptom severity module”)			
		Group 3 (−FA): 6-food ED			c. Exploratory proteomic and immune analysis			
		Group 4 (-FA): PO budesonide (1–2 mg based on weight)						
Heine [24]	Randomized, 2 arm	Group 1: oral PPI × 8–12 weeks + 4 food elimination diet	PPI alone	Histologic evidence of EoE expressed as #Eo/HPF	1. Mechanistic investigations regarding regulation of inflammatory and remodeling changes (eotaxins, TSLP, fibrosis markers, other related effector molecules)	N/A	N/A	
		Group 2: PPI alone × 8–12 weeks			2. Clinical response (symptom score)			
					3. Endoscopic appearance (endoscopy score)			

Of the studies measuring swallowed steroid, two assessed swallowed fluticasone, while two assessed swallowed budesonide. The three studies comparing swallowed steroid to placebo found that swallowed steroid was effective in improving histologic features [18, 20, 23]. One of those studies found that swallowed oral viscous budesonide was effective in improving symptoms in addition to endoscopic features. Schaeffer compared systemic and topical steroids and found that both were effective in achieving histologic and clinical improvement [19]. Although prednisone seemed to lead to a greater histologic effect, there was no difference observed between systemic and topical steroids in symptom resolution, relapse rate or time to relapse.

Of the two intravenous anti-IL5 trials, both found this agent to reduce intraepithelial esophageal eosinophilia. Spergel et al. found that while intraepithelial esophageal eosinophilia improved with treatment, symptom improvement was observed in all treatment groups, including placebo, and was not associated with changes in the esophageal eosinophilia [22].

Of the 11 identified studies, only 6 were published manuscripts, with relatively low risk of bias overall (see Table 8). Unfortunately intervention and controls were not sufficiently homogenous across studies to allow for comparisons. Meta-analysis in this case is unlikely to provide meaningful data, even across 3 or 4 swallowed steroid trials, or 2 anti-IL 5 studies. A meta-analysis for EoE treatment efficacy was considered; however, given the small number of studies, and heterogeneity in intervention type and outcome measures, this was not feasible.

**Table 8:** Risk of bias assessment in studies

	Konikoff [18]	Schaefer [19]	Dohil [20]	Assa’ad [21]	Spergel [22]	Gupta [23]
Random sequence generation	+	+	+	?	+	?
Allocation concealment	+	+	+	+	+	+
Blinding of participants/personnel	+	–	+	+	+	+
Blinding of outcome assessment	+	+	+	+	+	?
Incomplete outcome data	–	+	–	+	+	+
Selective outcome reporting	?	?	+	–	?	+
Pharmaceutical sponsorship	+	+	+	–	–	–

+ Low risk of bias

−High risk of bias

? Unclear risk of bias

## Discussion

This systematic review is one in a series of systematic reviews in the PORTal (primary outcomes reporting in trials) initiative, led by Dr. Vohra [11]. In PORTal, RCTs are evaluated to assess how well they report information about primary outcomes and outcome measurement instruments. This systematic review used the PORTal approach to examine these issues in pediatric EoE.

This systematic review identified a handful of exclusively pediatric EoE treatment trials. A number of outcomes were selected and reported in these trials, with certain measures, such as esophageal eosinophilia, clinical symptoms, safety, histologic features, and endoscopic features, re-occurring frequently, but not universally. The rationale for selecting outcome measures, and the measurement properties of the outcome measure tools (when used), were most often not reported. Based on the identified studies, no conclusions regarding treatment efficacy could be made.

### Clinical implications

Prior SRs of EoE treatment suggest a paucity of high quality evidence supporting current treatments for this condition, which, at this time include steroids, immune modulators, dietary modulation, mast cell stabilizers and esophageal dilatation [29, 30]. Indeed, much of the pediatric management guidelines for EoE are based on expert opinion and lower quality sources of evidence, such as retrospective observational studies, case reports, and case series [31]. Our current SR of pediatric trials confirms the need for additional treatment RCTs on the topic.

A recent prior SR examined EoE treatment efficacy in RCTs of children and adults up to the year 2010 [29]. While this review demonstrated heterogeneity in outcome measures and disease definition, this was not the focus of the review and these issues were not investigated thoroughly. Furthermore, that review combined adult and pediatric data, neglecting to account for the potentially significant differences in disease presentation, response to treatment, and outcomes, between adult and pediatric populations.

A 2014 SR of dietary treatment for EoE found that dietary interventions are effective in producing histologic remission in patients with EoE [30]. However, Arias et al. combined pediatric and adult data, and included observational studies and case series.

This is not the first time the question has been raised as to whether pediatric and adult EoE are manifestations of a single entity, or two distinct diseases [32, 33]. Notably, pediatric presentations of EoE have been noted to be more heterogeneous, and age-dependent, while in adults or older adolescents, the clinical presentation tends to be dominated by dysphagia and food impaction [16, 32, 33].

Controversy regarding the most pertinent end points in EoE trials has arisen before [34]. A 2011 editorial by Hirano noted that symptoms and histopathology on endoscopy (generally tissue eosinophilia) have been the most widely used outcomes. The editorial questions, however, whether, indeed, eosinophilia is an adequate or relevant outcome to measure in EoE trials, and urges for the validation of a patient-reported outcome instrument. Our results also find that eosinophilia is the most frequently selected outcome in pediatric trials.

The heterogeneity across outcome measures, and even within individual outcome measures, is not new in this field. Indeed, a 2011 editorial noted that even within commonly used outcomes, such as esophageal eosinophilia, there is variability in the methodology used to quantitate the eosinophils, as well as in the criteria for defining histopathologic change (e.g. reduction in peak eosinophilia versus number of eosinophils per high power field) [34].

Since EoE is being conceptualized as a clinicopathologic disease, experts in the field have emphasized the need for both symptoms and histology to be considered in any therapeutic trial [35]. Some researchers have already suggested that esophageal eosinophilia alone is not a sufficient trial primary end point. Fiorentino et al. suggested using clinical outcome assessment tools such as patient reported outcomes, where possible, with esophageal eosinophilia as a co-primary end point [36].

Significant strides have already been made within EoE research community, in order to address issues of outcome heterogeneity, and the complexity of disease definition for a disease where understanding continues to emerge. Recent American College of Gastroenterology (ACG) guidelines confirm the need for a combination of symptom and pathologic improvement as treatment end points [37]. The development and validation of a novel patient-reported outcome measure of dysphagia in patients with EoE is to be lauded [38].

However, it must be pointed out that a major limitation in the selection of appropriate outcome measures in pediatric EoE trials is our still evolving understanding of the natural history of this disease. Although consensus groups have urged investigators to select and report relevant outcomes, there is still no “gold standard” outcome measure for this disease. Clinical symptoms and histopathologic findings may both turn out to be important for diagnosis and outcomes, as these consensus groups have suggested, but the relative importance of each is not well defined.

Until more information is available regarding the natural history and pathophysiology of the disease, we would suggest that optimal studies in the field should present both clinical and histopathologic data and outcomes. Our systematic review demonstrated heterogeneity in outcome measures and disease definition, and will specifically guide pediatric EoE researchers who aim to design high quality pediatric RCTs in the future.

The uptake of the 2006 FIGERS criteria (50 %) in pediatric trials in our review is disappointing. Nevertheless, standardization of disease definition across clinical trials is a laudable goal. Another group previously investigated whether 2006 consensus guidelines for EoE diagnosis impacted diagnostic criteria reporting in the literature [39]. They found a significant increase in this reporting in articles published after the release of guidelines compared with those published earlier (31 vs 6 %, P < 0.001).

Of the 4 studies in our SR initiated after the new 2011 guidelines, only 1 adhered to the recommendations. Failure of 8 weeks of a PPI was not a requirement for 3 of the 4 studies. These studies may inadvertently be examining a more heterogeneous population than expected, including patients with PPI-responsive esophageal eosinophilia. Not consistently including or standardizing presenting symptoms of EoE as part of the disease definition makes looking at “clinical symptoms” as an outcome measure challenging. In some cases, patients did not have significant symptoms at baseline, or their symptoms varied dramatically at baseline within and between studies. This limits generalizability and comparability of results across studies, since studies included patients with varying degrees of disease severity. Not adequately defining symptoms at the start of the study might also negatively impact on ability to detect change over time.

Similar to the Cochrane review conducted by in 2010, in our SR, no meta-analysis could be conducted due to the limited number of heterogeneous trials identified [29].

### Limitations

The relatively low yield of studies in this systematic review may be related, in part, to the fact that only trials with exclusively pediatric participants were sought. By limiting included studies to those only of children, we optimized the likelihood of age-appropriate outcome measurement instruments being identified. If a study had a mixed adult and pediatric population, it is more likely that outcome measures used may be valid and reliable in one population but not in both (this would potentially disadvantage these studies in our assessment). In addition, there are data to suggest that symptoms, implications, and prognosis of EoE vary between children and adults [32, 33].

### Research implications

Primary and secondary outcome measures selected for the study of EoE varied considerably across treatment trials. No single outcome measure was selected and reported in all trials, which impedes knowledge synthesis. Furthermore, even for outcome measures frequently used (e.g. esophageal eosinophilia, clinical symptoms), standardized methods regarding how and when to assess them were lacking.

In the case of a relatively new and unstudied disease like EoE, validated measurement tools are lacking, which might partly explain the lack of validated measurement instruments being used in the present studies. Effort should be focused on validating measurement tools for use in the pediatric EoE population for future studies. Rationale for selection of outcome measures and appropriate references were rarely provided in these studies.

There is growing data showing that attention to standards for reporting in trials, as in the CONSORT initiative, leads to higher quality RCT reporting [8]. EoE is a relatively new condition and will benefit from research to evaluate which therapies are most effective. Future research would benefit from consistent and standardized definitions of disease occurrence and resolution, and from the development of a core outcome set so that investigators can agree on what outcomes to measure, when, and how.

## Conclusions

The results of this systematic review confirm the need for a core set of standardized pediatric outcome measures that are valid and reliable for future EoE trials. A standardized and rigorous approach to outcome measure selection, such as the COSMIN criteria would be appropriate. Adherence to standardized disease definitions will enhance future knowledge synthesis. Identifying and promoting resolution of heterogeneity in the definition of EoE and its resolution, as well as addressing the issue of heterogeneity in EoE RCT outcome measures, is critical to meaningful knowledge synthesis.

## Abbreviations

EoE: eosinophilic esophagitis
Eo: eosinophil
SR: systematic review
ACG: American College of Gastroenterology
